# Synchronous international scientific mobility in the space of affiliations: evidence from Russia

**DOI:** 10.1186/s40064-016-2127-3

**Published:** 2016-04-19

**Authors:** Yulia V. Markova, Natalia A. Shmatko, Yurij L. Katchanov

**Affiliations:** American Association for the Advancement of Science, 1200 New York Ave NW, Washington, DC 20005 USA; National Research University Higher School of Economics (HSE), 20 Myasnitskaya Ulitsa, Moscow, Russia 101000

**Keywords:** Affiliation, Bibliometrics, Scientific mobility, Scientometrics, Sociology of science

## Abstract

The article presents a survey of Russian researchers’ synchronous international scientific mobility as an element of the global system of scientific labor market. Synchronous international scientific mobility is a simultaneous holding of scientific positions in institutions located in different countries. The study explores bibliometric data from the Web of Science Core Collection and socio-economic indicators for 56 countries. In order to examine international scientific mobility, we use a method of affiliations. The paper introduces a model of synchronous international scientific mobility. It enables to specify country’s involvement in the international division of scientific labor. Synchronous international scientific mobility is a modern form of the international division of labor in science. It encompasses various forms of part-time, temporary and remote employment of scientists. The analysis reveals the distribution of Russian authors in the space of affiliations, and directions of upward/downward international scientific mobility. The bibliometric characteristics of mobile authors are isomorphic to those of receiver country authors. Synchronous international scientific mobility of Russian authors is determined by differences in scientific impacts between receiver and donor countries.

## Background

For the sociology of science, bibliometric data acts as a kind of ‘footprint’ recording the digital signatures of scientific processes. However, this footprint does not in itself convey sociological content (see for example Mingers and Leydesdorff 2015). Bibliometric databases such as the ‘Web of Science Core Collection—Thomson Reuters’ (hereafter—WoS CC) provide an opportunity to investigate a wide range of social scientific phenomena, but place the sociology of science within the strict limits of information technology. If the sociologist wants to avoid the bibliometric data on the screen becoming like the wall of Plato’s Cave, he must learn how to work with relatively poor, formalized information. Fortunately, modern sociology of science’s perspective on mobility is sufficiently broad to allow the use of digital traces of scientists’ movements, using fixed WoS CC affiliations.

The term ‘scientific mobility’ denotes the movement of scientists from one position in social space to another (cf. Bourdieu 1985; Shmatko and Katchanov 2016). Generally, this space is comprised of a network or structured system of socially defined positions. Social space ‘is constructed in such a way that the closer the agents, groups or institutions which are situated within this space, the more common properties they have; and the more distant, the fewer’ (Bourdieu 1989, p. 16).

The concept of scientific mobility deals with the movement of scientists from one position to another, either higher or lower in social space. Scientific mobility contributes both to the continuity and change of social space over time. In basic terms, we can say that scientific mobility analysis is the study of social shifts which have a synthetic (i.e. composite) structure. Since scientific mobility entails science-sustaining and academia-sustaining core transformations, answers to fundamental questions about social features, horizons, and opportunities of science and academia depend on the correct specification of that mobility.

In the study of scientific mobility through bibliometric methods, institutional affiliation is an important (and indeed, often the only) criterion for membership of a social position. This situation is easily explained, because the affiliation has a synthetic character, closely linked with other aspects of a scientist’s status. In classical sociological studies of social mobility profession is used as a main tool to construct social positions. In our case, affiliation plays the same role. The realm of institutional affiliations contains information about an important aspect of scientific mobility: international scientific mobility (or ISM for short). Referring to the above definition of scientific mobility, in the case of ISM we can regard social space as a kind of geopolitical space, which is demarked by national territorial boundaries.

ISM begins to be actively studied when it becomes a widespread phenomenon, and thus a subject of interest in public policy. In recent times the term ‘brain drain’ has emerged, to refer to the unidirectional migration of scientists from less developed to more developed countries (Report of a committee appointed by the Royal Society 1963). However, the evolution of global scientific production has led to the formation of a more complex system of scientists’ mobility between countries (Ackers and Gill 2008; Van Noorden 2012; Gargiulo and Carletti 2014), scientific organizations (Deville et al. 2014a) and sectors of production (Edler et al. 2011). The main factors behind this transformation are as follows (Fernández-Zubieta et al. 2015):

- changes in migration legislation;
- the creation of student exchange systems;
- mutual recognition of diplomas among countries;
- the spread of new means of communication;
- the creation of large international scientific organizations;
- the expansion of international collaboration networks.

All this has led to a situation where ISM is being perceived less as ‘brain drain’, and more as a form of ‘brain circulation’ (Gaillard and Gaillard 1997; Jöns 2009; Li et al. 2015). Other aspects of mobility include temporary mobility of researchers (Cañibano et al. 2011; Lawson and Shibayama 2013), the emergence of national scientific diasporas (Barré et al. 2003; Chikanda et al. 2016; Marmolejo-Leyva et al. 2015; Seguin 2006), and the return of mobile researchers to their home countries (Andújar et al. 2015; Jonkers and Cruz-Castro 2013). Special attention is given to the mobility of Chinese researchers Jonkers (2010) and Lu and Zhang (2015). The trend of scientific migration to the United States persists: many researchers strive to move to the USA rather than other countries (Harvey 2011; State et al. 2014; Veugelers and Van Bouwel 2015; Wang et al. 2013).

ISM has ambiguous consequences for both receiver and donor countries. In the current structure of global science it is not always easy to assess who the winners and who the losers are Beine et al. (2008) and Docquier and Rapoport (2012). For example, the active migration of researchers to the USA affects salary levels in science and leads to the growth of temporary contracts (Stephan 2012), while the return of scientists from overseas contributes to the development of science in their home countries (Ding et al. 2010; Saxenian 2005).

We should like to remark that it is not our intention to present here a complete review of ISM. The subject is so vast that it is impossible to include every important contribution, and there are many reviews, which do this. Additional bibliographic discussions and references can be found in the books Ackers and Gill (2008), Archibugi and Filippetti (2015), Geuna (2015) and Solimano (2008).

Russian scientific migration and mobility are rather well studied. There are a significant number of studies on the impact of migration on the development and productivity of Russian science Latova and Savinkov (2012) and Shkolnikov (1994), as well as studies on the mobility of researchers in particular fields (Borjas and Doran 2012a; Boussyguine 2005; Indukaev et al. 2014) and scientific diasporas Graham and Dezhina (2008). To avoid overloading our presentation, we refer the reader to Charum and Meyer (1996), Ganguli (2015), Gerber and Yarsike Ball (2009), Ivakhnyuk (2006) and Korobkov and Zaionchkovskaia (2012) for details.

## Method of affiliation as a means of studying ISM

ISM refers to scientists undertaking professional activities in scientific organizations which are located in different countries. Traditionally, ISM is studied using such methods as government statistics on academic staff and data on migration (Arvizu and Bowen 2014; OECD 2013), analysis of CVs and personal web pages (Cañibano and Bozeman 2009; Sandström 2009; Woolley and Turpin 2009), questionnaires and interviews with scientists themselves (Boring et al. 2015; Flanagan 2015; Gerber and Yarsike Ball 2009), and government and administrative databases (De Filippo et al. 2009). The bibliometric approach to the study of scientific mobility is based on the information about institutional affiliation which is included in any scientific publication. We call it the ‘affiliations method’. A scientist’s place of work is like a ‘data tag’ indicating his location in the social space (country, city, organization). The websites of academic journals and databases of scientific publications serves as sources of information on institutional affiliations. The main ones are WoS CC and Scopus (Elsevier). Alongside these, such databases as Pubmed, Inspec, Mathnet and others may be used.

By studying the distribution and trends in institutional affiliations, one may track the trajectory of individual scientists (for example through the profile of the author, as on the Scopus database), and analyze mobility at the level of academic groups, individual disciplines and countries (Dubois et al. 2014; Moed and Halevi 2014; Moed et al. 2013). This method makes it possible to carry out a comparative study of the publishing activity of mobile and non-mobile authors Pierson and Cotgreave (2000), and to study the impact of migration on the development of various disciplines Borjas and Doran (2012b). By using the affiliations method, it is also possible to study the mobility of groups of elite scientists that are small, but nonetheless important for the development of science (Laudel 2005). These ‘digital traces’ can capture the movement of scientists between countries (Furukawa et al. 2011, 2012); the concentration of representatives of various disciplines in certain countries or organizations (Deville et al. 2014b); and enable analysis of relative migration flows (Moed and Halevi 2014). There are a range of studies on the ISM in individual countries, such as Canada and Germany (Conchi and Michels 2014; Roberge and Campbell 2012), Argentina, Colombia and Uruguay (Meyer et al. 2016), as well as comparative studies on a range of countries (Moed and Halevi 2014; Moed et al. 2013). Thus, the affiliations method enables the study of different types of researchers’ movements in the space of affiliations (Table 1).

**Table 1:** Types of author movements in the space of affiliations

	Diachronic mobility	Synchronic mobility
Organization	Mobility from one organization to another	Multiple organizational co-affiliation
Country	Migration from one country to another	Multiple international co-affiliation
Sector	Move from university to enterprise, from university to research institute, etc.	Being simultaneously affiliated with university and enterprise, with university and research institute, etc.

However, all of these studies consider *diachronic* ISM, i.e. the movement of scientists from one country to another, and disregard *synchronous* ISM—simultaneously holding scientific positions in organizations located in different countries. For instance, in their study of the contribution of different countries to the production of publications in the field of high energy physics, Kraus, Mele and Lindqvist categorize authors with multiple affiliations as belonging to just one country (Krause et al. 2007). We find similar approach in OECD studies and some others (OECD 2013; Quayle and Greer 2014). Yet, such synchronous mobility is quite common. The bibliometric method records this form of mobility as multiple affiliations. Synchronous international mobility of individual scientists serves as a marker of the extent to which national scientific labor markets overlap, and the strength of international relations between countries.

ISM is one of the paradigms in the sociology of science and scientometrics. New approaches in scientometrics often arise not from discarding current paradigms, but rather by expanding them to provide more substantial information. In this article we have expanded the paradigm of ISM by studying the synchronous international scientific mobility of Russian researchers. Since ISM is a global structure, its components should be investigated in their synchronous relationships. Needless to say, the evolution of ISM means that new synchronous relationships are continually being generated. However, the core patterns of the ISM system can only be identified by means of synchronous analysis. Analysis of synchronous international scientific mobility (SISM for short) as the aggregate of permanent statistical relationships between affiliations reveals the structure of ISM. This structure is presented in our paper as the space of affiliations.

In the present paper, we focus our study on SISM in the framework of multiple affiliations. We aim to characterize SISM through study of the statistical relationships between separate institutional and geographical movements of Russian authors. Our purpose is to understand which factors determine the space of affiliation.

## Information and data sources

The empirical object of our analysis is a sample of papers by Russian authors published between 2008 and 2013 and indexed in the database WoS CC (access date: June 2014). Six years is a sufficient publication window to obtain an adequate sample of papers that is resistant to stochastic fluctuations.

We classified a publication as ‘Russian’ if at least one of the authors had a Russian institutional affiliation, i.e. their place of work was listed as an organization located in Russia. Russian authors published a total of 199,965 articles and other types of document during the period under analysis. On average, the WoS CC database indexed 33–34 thousand publications by Russian authors annually.

To characterize countries in the space of SISM we use the following data sources:

- Indicators of socio-economic and scientific development of countries provided by the UNESCO Institute for Statistics (access date: 19.10.2015).
- The Human Development Index, created as part of the United Nations Development Programme (access date: 19.10.2015).
- Indicators of scientific productivity and the level of scientific influence of individual countries, as given in the Thomson Reuters ‘Essential Science Indicators’ database (access date: 19.10.2015). We based our study on a sample of 56 countries. This included the 50 countries that had the highest number of publications over the period 2008–2012 in the ‘Essential Science Indicators’ database. In order to analyze scientific relations between Russian scientists and their colleagues from the former USSR, former Soviet countries were also included in the sample. The resulting sample of countries was used in the study that follows.

The sample of Russian publications includes just over 353 thousand authors. However, many authors have just one or two publications for the entire period. We can assume that they are either foreigners who came to Russia for a very short period of time, or scientists who left their academic career. In order to eliminate any confounding effects of such cases, we introduced a ‘productivity filter’, which allowed us to establish a body of *active authors* (hereafter AA). AA included authors who produced at least three publications during the study period, i.e. the *productivity threshold* was a minimum of three publications in 6 years. In this way we formed a sub-sample of just over 71 thousand authors to serve as the basis for further analysis.

In studying SISM through bibliometric methods, we determine an author’s country on the basis of their institutional affiliation during the publication window of 2008–2013. The author is considered a scientist of a given country if he or she has an institutional affiliation in that country in the sample of publications. In our sample of Russian publications, this method allows to distinguish between three main groups of authors:

- Russian—authors who were only affiliated with Russian organizations in the study period, i.e. scientists who had no professional positions outside the country (hereafter RA).
- mobile—authors who were affiliated with at least one Russian organization and any number of non-Russian organizations during the study period (hereafter MA).
- foreign—authors who were not affiliated with any organization located in Russia during the entire study period (hereafter FA). These are foreign co-authors of Russian scientists.

Let us consider the structure and trends in the distribution of the AA by the groups RA, MA, FA. During the period under analysis, the share of MA remained constant at just under 10 %. For comparison, Conchi and Michels found that the proportion of MA was 8 % in both Germany and Austria, 6 % in France, and 7 % in the UK (Conchi and Michels 2014).

In the Russian case AA had an average of 36 % FA, and their number gradually increased (from 35 to 39 %). On the one hand this is hardly surprising, in view of the increasing number of publications with a large number of co-authors, especially in the field of physics, which accounts for a significant share of Russian publications. On the other hand, it suggests that the Russian publication activity of AA depends essentially on collaboration with scientists from other countries. For comparison, Conchi and Michels found that the share of FA in Germany and Austria is 10, 7 % in France, and 12 % in Great Britain (Conchi and Michels 2014).

In our sample MA accounts for around 15 %, and RA 85 % for the entire period of study. MA totals 6249 authors. This statistical group serves as the empirical basis for our further analysis of SISM.

## Concept of SISM

The emergence of one or more foreign affiliations corresponds to researcher’s mobility in geographical spaces. Hence, synchronous international scientific mobility (SISM) can be defined as the emergence and loss of foreign affiliations. The SISM model considers that the researcher participates in each of the institutes with which she/he is affiliated not as a unified whole but only as a part; the latter we call ‘parton’. A parton has a virtual bibliometric identity that appears in processes of international division and cooperation of research if these processes are de jure formed via multiple simultaneous affiliations. As noted above, our sample counts 6249 MA, which constitute 10,826 partons. According to the SISM model, a mobile researcher behaves as a combination of partons. The multiple affiliations in the framework of bibliometrics are to a some extent uncertainty in the sense that one cannot predict exactly where the scientist will be, but only the probability that she/he is in a country corresponding to one of several partons of this author. Therefore, it is necessary to include probability when describing SISM. Analogous to quantum mechanics, we only can estimate the probability that a researcher with multiple affiliations is located in a given country.

If we are talking about multiple affiliations, then the author is ‘delocalized’: his/her characteristics are distributed in both the geographic space and in the space of research institutes. Moreover, any co-authored paper is a result of a process often involving a significant number of researchers, each of whom may have multiple affiliations. That yields that the local description of every research paper is effaced. The concept of SISM provides us with a deep insight into spatially distributed information interactions, because bibliometrics does not give us sufficient information to be able to uniquely determine the location of an author. In conventional ISM the scientists are ‘mathematical points’ in space and time, whereas in SISM the fundamental objects are consistent sets of partons, and the ‘intensity’ of the parton in a country gives probability of finding the scientist in this country. The main idea of SISM is that instead of an accurate tracking of geographic location and institutional affiliation depending on time, we can evaluate the probability that a researcher is located in a given state. This probability can be used to calculate statistical indicators of SISM.

**Fig. 1:**
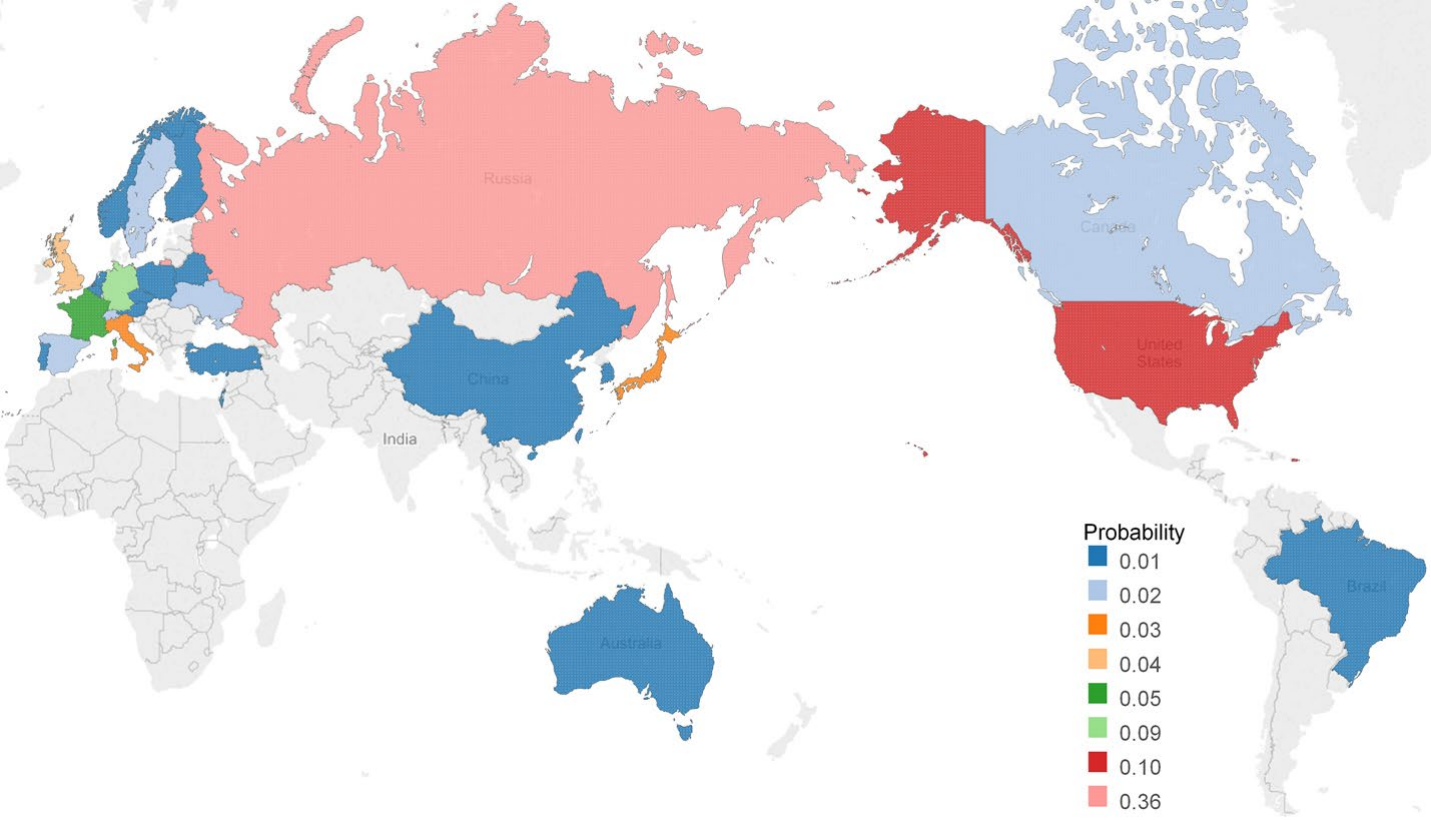
The probability of a parton having an affiliation in a given country. Key. The map shows the countries for which the probability that parton is affiliated with the country is greater than or equal to 0.01. This includes 28 countries: USA, Germany, France, United Kingdom, Italy, Japan, Canada, Spain, Sweden, Switzerland, Ukraine, Australia, Austria, Belarus, Belgium, Brazil, China, Czech Republic, Finland, Israel, Netherlands, Norway, Poland, Portugal, South Korea, Taiwan, Turkey

Figure 1 shows the probability of a parton ‘to be affiliated with a given country’. For our sample the probability of having an affiliation in Russia is 0.36, for the USA—0.10, for Germany—0.09, for France—0.05, for Great Britain—0.04, for Italy and Japan—0.03. As for Canada, Spain, Sweden, Switzerland and Ukraine the probability is 0.02 for each country. For the other countries in our sample, it is 0.01 or less.

The initial concept in the SISM approach is an event of affiliation. The events of affiliations form, in bibliometrics, the mass phenomena comprising the subject of SISM. By event of affiliation, we understand any fact of affiliation, which in certain circumstances can occur or not. As a bibliometric concept, an event of affiliation in the framework of SISM is only defined in terms of whether it occurred or not, but not by its concrete social nature. Bibliometrically, an event of affiliation is seen as a state of the corresponding researcher.

To bibliometrically describe SISM, we first need the notion of the space of affiliations corresponding to the data set under consideration. The space of affiliations is an aggregate of events of affiliation. The concept of an affiliation set of partons is the basis for the practical definitions of the space of affiliations. We say that a homogenous collection of partons is an affiliation set, if it contains all events of a given affiliation. In this case, a space of affiliations is a family of affiliation sets (constructed statistically) on condition that there are objective relations within this family similar to the usual spatial relations. Such a system of statistical positions can be likened to a chessboard: all the significance arises from differences between the positions.

If we take country affiliation as a characteristic affiliation, then we are able to describe the family of affiliation sets in terms of political geography. In bibliometrics, the space of affiliations plays a role of a space of existence and development of forms of scientific communication. As a prerequisite, the space of affiliations manifests itself in international scientific cooperation.

In this paper, we construct the space of affiliations based on the following two types of relations:

1. Relations of membership and inclusion: an event of affiliation belongs to the affiliation set, and one affiliation set is contained in another. In actuality, we base our hypotheses about the space of affiliations on the fact that any affiliation set is composed of events of affiliation. Events of affiliation differ above all by their belonging to one or another affiliation set. Events included to one affiliation set are seen as identical. We embed separated events of affiliation into affiliation sets, and statements of these separate events are replaced by statements of their statistical aggregate.
2. As soon as we divide events of affiliation into affiliation sets, and these events themselves are characterized by their membership to a given affiliation set, then it is necessary to describe the relations between these sets. Distance characterizes spatial relations between affiliation sets. In this way, constructing the space of affiliations builds on the general understanding of distance. The space of affiliations is a family of affiliation sets in which the rule for measurement of distances is well-defined.

## Vertical mobility

In order to rank the SISM of countries we consider their socio-economic characteristics. The following indicators are used as markers of the level of countries’ development:

- GDP (Gross Domestic Product)—an indicator of the level of economic development of a country (in 2008);
- GERD (Gross domestic expenditure on research and experimental development)—an indicator of the level of funding for research and development in a country (in 2008);
- RDP (R&D personnel in full time equivalents)—an indicator of the level of the stable work market in a country (in 2008);
- HDI (Human Development Index)—indicator of achievements in key dimensions of human development (in 2008);
- NP (number of papers)—an indicator of the impact of a country’s science (in 2008–2012, according to data from Essential Science Indicators);
- NC (number of citations in 2008–2012)—an indicator of the level of influence of a country’s science (according to data from Essential Science Indicators);
- NTP (number of top papers in 2008–2012)—an indicator of the level of influence of a country’s science (according to data from Essential Science Indicators).

Indicators of the SISM of Russian scientists:

- PA (the number of partons having an institutional affiliation to a particular country)—an indicator of Russian authors’ degree of mobility to this country;
- PP (the number of publications by partons collaborating with a given county)—an indicator of the level of publishing activity of partons;
- PC (the number of citations of partons collaborating with a given county)—an indicator of the level of scientific influence of partons.

A correlation analysis of this system of indicators revealed a number of important patterns in the SISM. Let us consider GDP, together with PA. The data shows that there is a significant positive correlation between them: the higher the figure for GDP, the larger the figure for PA. The Pearson’s correlation coefficient for GDP/PA is 0.812 (Table 2).

**Table 2:** Pearson’s correlation coefficient between PA and countries’ indicators

Indicator	*r*	*p* value
GDP	0.812	0.000
GERD	0.758	0.000
RDP	0.533	0.000
NP	0.804	0.000
NC	0.837	0.000
NTP	0.822	0.000

Of 6249 MA, 89.7 % maintain professional scientific ties with countries in which the HDI is higher than in Russia. These are Russia’s main scientific partners: USA, Germany, France, Japan and Italy, together with three other countries: Norway, Australia and Switzerland.

We now turn to indicators which allow us to assess the level and scale of the development of science in the country (GERD, RDP, and NP). The relationship between the three indicators and PA is analogous to its relationship with GDP: PA displays a significant positive correlation with the three indicators (Table 2). We can say the same about PA, NC and NTP. There is a strong positive correlation between PA and the two indicators of a country’s scientific impact. From this we can conclude that PA focus on countries where science is better funded and where there are more opportunities for carrying out large-scale and long-term studies. In turn, the development of scientific production creates a labor market that acts as an attractor for PA.

The correlation between NP and PA allows us to conclude that countries with high level of performance attract PA. However, it should also be noted that on average MA have a higher level of publication activity than RA. MA have an average of 54 publications, while RA have an average of just 9 (Table 3). At the same time, PP correlates well with NP (*r* = 0.783).

**Table 3:** Indicators of publication output of mobile and non-mobile Russian authors

	Number of papers		Number of citations	
	Mobile	Non-mobile	Mobile	Non-mobile
Mean	54	9	1102	43
Median	10	6	59	9
Standard deviation	99.4	13.74	2308.55	291.36

The correlation between NC, NTP and PA suggests a similar trend: the higher the level of scientific impact of the national science, the higher PA it attracts. In turn, the level of citations of MA is significantly higher than for RA (Table 3). Our results accord with the OECD’s data for other countries: the “impact factor” of mobile authors is higher than that of non-mobile authors OECD (2013). In addition, according to the citations indicator, the citation rate for mobile authors’ publications is approaching that for authors of the receiver country. There is a strong positive correlation between PC and the level of citations, NC ($r = 0{.}834$). In terms of publication activity and citations, the relationships between MA and RA on the one hand, and FA and Russian authors (as a whole) on the other hand, are isomorphic. That is to say, MA have higher publication activity (and more citations) than RA, just as authors from the leading scientific nations have higher publication activity (and more citations) than all Russian authors, including MA.

A significant statistical relationship between countries’ socio-economic and scientific indicators and PA points out that SISM acts as a means of virtually ‘drawing in’ partons to the scientific systems of more developed countries. (In contrast to the analysis of co-authorship, which often does not establish direct links between countries, the study of SISM allows us to show the direction of close scientific cooperation and the place of a country in the international division of scientific work.) The higher the economic and scientific indicators of a country, the more partons the country can integrate into its scientific system. In the leading scientific countries partons usually operate as temporary, part-time and remote workers. They are the most flexible part of the scientific labor force. In turn, partons have an impact on the scientific labor market of their host country, expanding the zone of social uncertainty, insecurity and flexible employment.

Affiliation with leading countries contributes to greater scientific productivity and heightened reputation for MA. In comparison with RA, MA receive more scientific recognition (as measured by citations) and more opportunities to carry out projects, which in turn contributes to their high rate of publication activity. In this respect, MA become like their foreign colleagues. Therefore, in terms of productivity and level of recognition, the gap between MA and non-mobile authors is similar to that between authors in the leading receiver countries and MA. This means that the bibliometric characteristics of MA are isomorphic to those of receiver country authors. This supports our thesis that SISM acts as a form of social mobility for Russian scientists.

For MA, international virtualization has mixed consequences. On the one hand, it extends the MA’s professional opportunities, as it allows heterogeneous scientific resources to be combined and compensates for the shortcomings of Russian science. Moreover, such involvement does not require the MA to relocate in geographic space; in many cases, partons are associated with a foreign research institution remotely, only pro forma, as part of a bureaucratic reporting etc. On the other hand, the existence of partons is associated with the uncertainty of social and professional status, the temporary and non-guaranteed nature of institutional positions.

In essence, SISM is a modern, virtual form of the international division of labor in science, to some extent supplanting real migration. Researchers seek work where it exists, even when it is in a partial and/or virtual form, and where there are greater opportunities to receive scientific recognition in the form of citations.

**Fig. 2:**
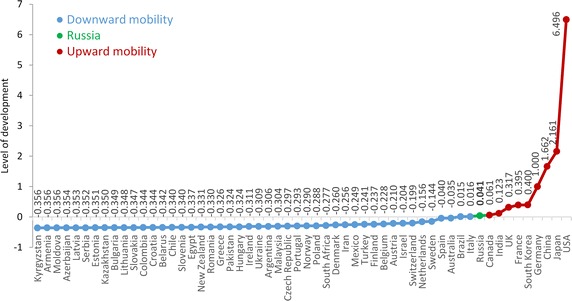
Level of countries’ development

To determine the one-dimensional configuration of our $56$ countries in the space of social-economic indicators, we used the ALSCAL procedure (Takane et al. 1977), found in IBM SPSS Statistics 20. In this paper, we interpret this configuration (see Fig. 2) in a broader sense as the variable that indicates the level of development of countries and the determinants of upward/downward SISM.

In the present paper, Russia serves as a reference point for the assessment of vertical SISM. Affiliations in countries that are located to the right of Russia on Fig. 2 provide upward SISM, while affiliations in countries to the left of Russia provide neutral or downward SISM. Countries located ‘above’ Russia are Canada, India, Great Britain, France, South Korea, Germany, China, Japan and the United States. These are either countries with developed science or those where it has been developing dynamically in recent years (India, South Korea and China). In other words, upward SISM is enabled by institutional links either with those countries that are the traditional leaders in science or those where scientific production is actively expanding.

**Fig. 3:**
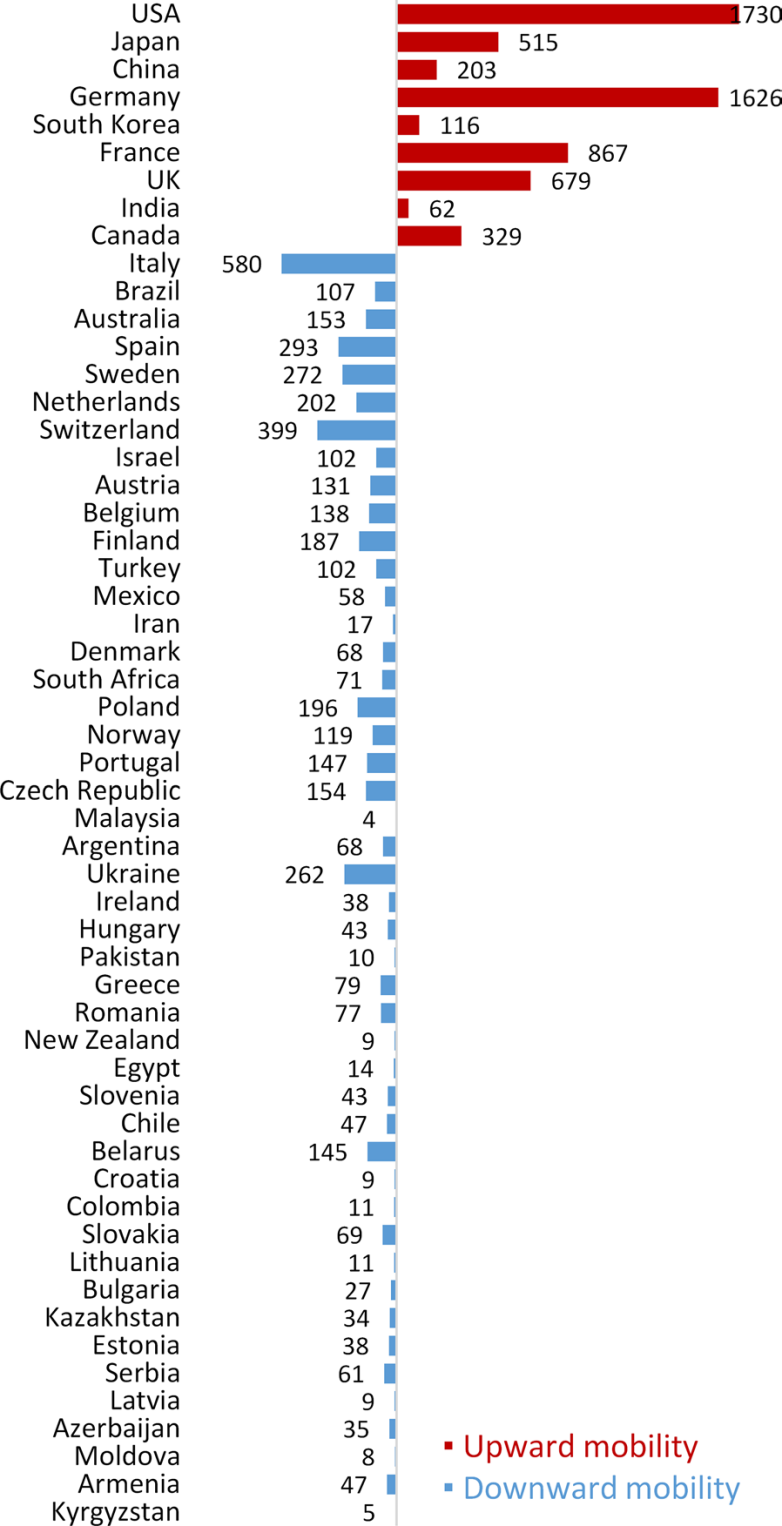
Upward/downward SISM and PA

Further to the left of Russia in Fig. 2 are the countries of the former Soviet Union (with the exception of Ukraine and Belarus), and former member states of the Council for Mutual Economic Assistance (except Poland). In these countries science is either stagnating, or contracting. Figure 3 shows PA in each country.

## Russian space of affiliations: between East and West

As a result of the information gathered from the WoS CC database, to each author we can assign a numerical code $\xi$, and $\eta$ is a numerical code that corresponds to each paper. Thus, $\xi$ is a unique identifier of an author, and $\eta$ is a unique identifier (or ID) of a paper, respectively.

The first object encountered in the SISM model is the space of elementary outcomes, or partons. Generally, it is a non-empty set $\Omega$, whose elements $\omega \in \Omega$ are partons, or sample points. Obviously, the specification characterizing a parton $\omega$ can, e.g., be: ‘the paper ID $\eta _{i}$, the author ID $\xi _{j}$, with Russian and German affiliation’. Roughly speaking, the value of $\omega$ attached to a paper is what we call the parton, the description of one of the authors of this paper at the adopted level.

In the framework of probability theory, an event of affiliations corresponds to a set of partons, i.e. any subset $A\subseteq \Omega$ is called an event of affiliations. If we define, e.g., the parton $\omega$ with Russian and German affiliation, it makes sense to ask whether $\omega \in A$, and to assign a certain probability

$$\begin{aligned} \hat{\mathrm {P}}_{n}(A) = \frac{\nu (A)}{n} \end{aligned}$$

to the event of affiliations $A$. Here $\nu (A)$ is the number of the partons which belong to $A$. The empirical distribution function is defined as $\hat{F}_{n}(x) = \hat{\mathrm {P}}_{n}\bigl ((-\infty , x)\bigr )$. Choosing certain double affiliation (i.e. Russia and given country), we can describe SISM to given country by an empirical distribution function $\hat{F}_{n}^{(i)}(x)$.

We consider the subspace $D_{e}$ of the Skorokhod space [see details in Jacod and Shiryaev 2003] containing all bounded empirical distribution functions. We can to provide the space $D_{e}$ with the Kolmogorov metric Rachev et al. (2013)

1 $$\begin{aligned} \varrho \left( \hat{F}_{n}^{(i)}(x), \hat{F}_{m}^{(j)}(x)\right) = \sup _{x}\left| \hat{F}_{n}^{(i)}(x) - \hat{F}_{m}^{(j)}(x) \right| . \end{aligned}$$

Furthermore, consider the matrix $R$ of pairwise distances Eq. (1), in the space $D_{e}$, between the $55$ countries

2 $$\begin{aligned} R = \left( \varrho _{ij} \right) _{i,j = 1}^{i,j = 55}, \end{aligned}$$

where the distance between two countries reflects their bibliometric differences. We use multidimensional scaling techniques (namely, the ALSCAL procedure) to derive country position in a space of affiliations that fit Kolmogorov distances Eq. (2). The space of affiliations is a spatial configuration of the $55$ countries, represented as points in a Euclidean space of two dimension. As a matter of fact, the structure of the space of affiliations reflects both the publication activity of MA, and the level of involvement of MA in the scientific systems of other countries.

**Fig. 4:**
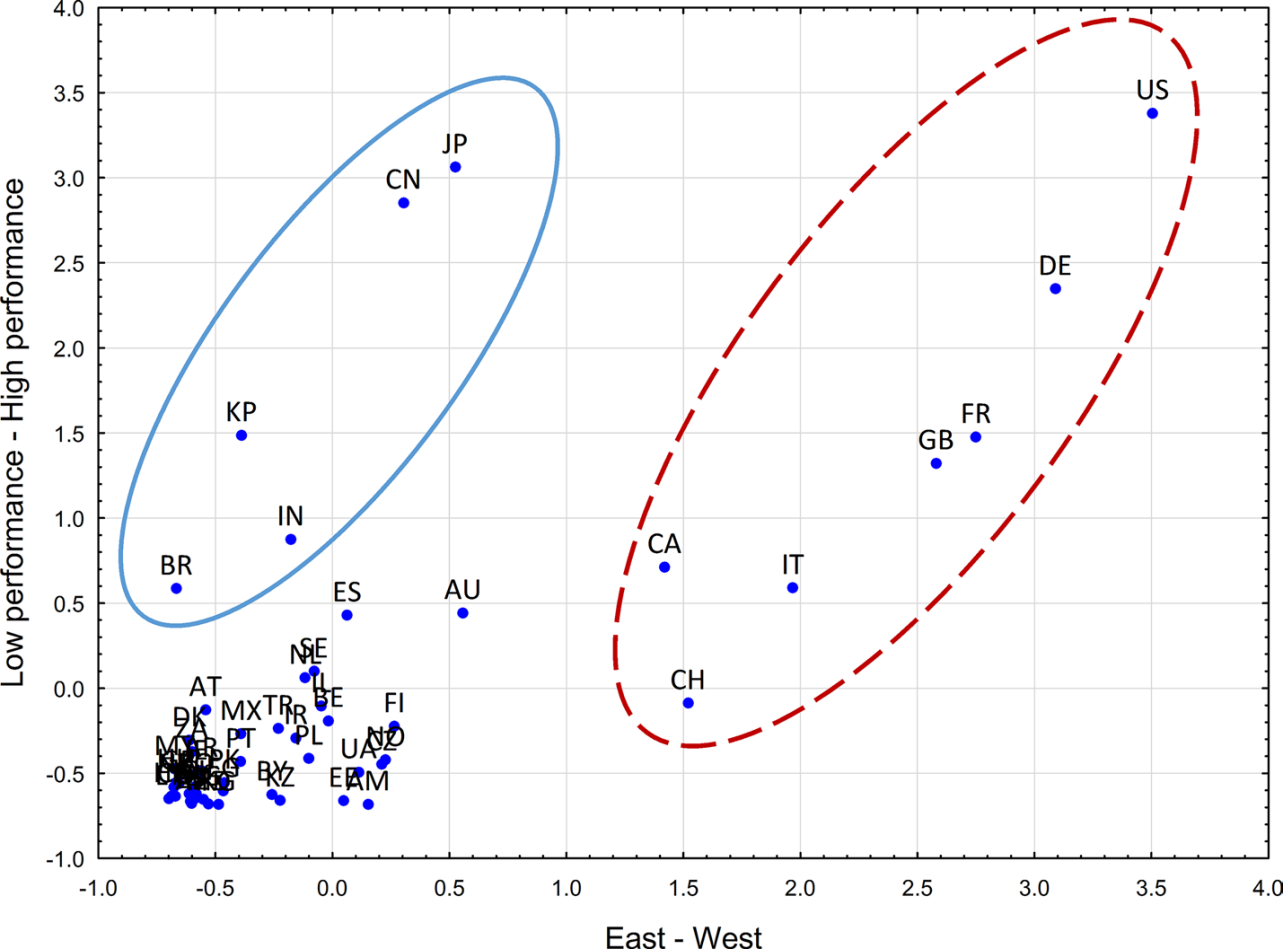
Countries in the space of affiliations. Key. *AR* Argentina, *AM* Armenia, *AU* Australia, *AT* Austria, *AZ* Azerbaijan, *BY* Belarus, *BE* Belgium, *BR* Brazil, *BG* Bulgaria, *CA* Canada, *CL* Chile, *CN* China, *CO* Colombia, *HR* Croatia, *CZ* Czech Republic, *DK* Denmark, *EG* Egypt, *EE* Estonia, *FI* Finland, *FR* France, *DE* Germany, *GR* Greece, *HU* Hungary, *IN* India, *IR* Iran, *IE* Ireland, *IL* Israel, *IT* Italy, *JP* Japan, *KZ* Kazakhstan, *KG* Kyrgyzstan, *LV* Latvia, *LT* Lithuania, *MY* Malaysia, *MX* Mexico, *MD* Moldova, *NL* Netherlands, *NZ* New Zealand, *NO* Norway, *PK* Pakistan, *PL* Poland, *PT* Portugal, *RO* Romania, *RU* Russia, *RS* Serbia, *SK* Slovakia, *SI* Slovenia, *ZA* South Africa, *KP* South Korea, *ES* Spain, *SE* Sweden, *CH* Switzerland, *TR* Turkey, *GB* United Kingdom, *UA* Ukraine, *US* USA

In Fig. 4 several clusters of countries can be clearly observed. On the right of the ‘East-West’ axis is a group of seven countries (Switzerland, Canada, Italy, Great Britain, France, Germany, and the USA). Almost all of them (with the exception of Switzerland) are members of the G7. In our terminology these are countries with upward SISM; in Fig. 4 they form a ‘Western’ pole. The Western pole accounts for $57{.}8\,\%$ of PA.

In the upper left hand area of the space of affiliations (see Fig. 4) are China and Japan, with South Korea, India, and Brazil lying just below them. These are predominantly countries with rapidly growing science, with the exception of Japan, which is one of the traditional scientific leaders. These five countries form an ‘Eastern’ pole in the space of affiliations, and account for $9{.}3\,\%$ of PA.

In the lower left hand corner of the space of affiliations is a large cluster of countries with low or relatively low levels of performance and citation impact. MA have less intensive scientific connections with these $43$ countries ($32{.}8\,\%$ of PA).

In the space of affiliations the bibliometric characteristics of MA are isomorphic with countries’ scientific characteristics: the higher and further right the MA is located, the higher on average their productivity and level of citations. Migration in the space of affiliations is possible only in accordance with the regularities of the citation system. In other words, to move from the bottom left to the upper right hand corner, an author needs to produce a large amount of scientific work, dramatically increasing their bibliometric indicators.

## Model of SISM

The task for the SISM model is to estimate the number of partons affiliated with each country. Although the mathematical formulation that follows is quite cumbersome (see Helbing 2010), the style of presentation is that of a non-mathematical text with language that is far from formal. Currently, the most successful models of social mobility are ‘push-pull’ models Weidlich and Haag (1988). This type of model represents individual probability transition rates $$v_{ij}$$ from $$i$$-th social group to $$j$$-th social group as a function $$\phi$$, firstly, of utility $$u_{i}$$$$i$$-th of group, secondly, utility $$u_{j}$$$$j$$-th of group, and thirdly, generalized social distance $$d_{ij}$$ between $$i$$-th and $$j$$-th groups (cf. Pan et al. 2012)

$$\begin{aligned} (i\ne j):p_{ij} \varpropto \frac{\phi (u_{i})\phi (u_{j})}{d_{ij}} . \end{aligned}$$

Consistent application of the stochastic system analysis in the framework of generalized rational-choice conception results in the Weidlich (2006) Weidlich–Haag model of social mobility, in which the individual probability transition rates take the following form

3 $$\begin{aligned} (i\ne j):p_{ij} \varpropto \frac{\exp \left( u_{i} - u_{j}\right) }{d_{ij}} . \end{aligned}$$

In this expression, the value of utility $u_{i}$ expresses the attraction of an individual to $i$-th social group, while the utility $u_{j}$ reflects the ‘pulling away’ of an individual from $j$-th social group. Moreover, individual probability transition rates are inversely proportional to the generalized sociological distance $d_{ij}$ between $i$-th and $j$-th groups.

Clearly, we can use, mutatis mutandis, the Weidlich—Haag model to assist our research. We will proceed by interpreting $j$-th social group as Russia, and $i$-th social group as the destination country. It is logical to take the share of received citations by country $k$ as the ‘utility’ gained by PA.

$$\begin{aligned} \gamma _{k} = \frac{NC_{k}}{\sum _{l = 1}^{55}NC_{l}} . \end{aligned}$$

In our case it is natural to identify the generalized distance $d_{ij}$ with Kolmogorov distance $\varrho _{ij}$ Eq. (1).

However, unlike the Weidlich—Haag model, we are not interested in individual transition probabilities $p_{ij}$, but rather probabilities of countries $\hat{\mathsf {P}}_{i}^{*}$. In the above notations, we can write the following expression for the $\hat{\mathsf {P}}_{i}^{*}$

$$\begin{aligned} \hat{\mathsf {P}}_{i}^{*} := \frac{\nu \left( {P\!A}_{i}\right) }{N} , \end{aligned}$$

where $N = 10{,}826$ is the general number of PA.

Taking into account Borovkov (2013), we obtain

4 $$\begin{aligned} (i\ne j):{\mathsf {P}}_{i}^{*} \varpropto \frac{\left( \exp \left( \gamma _{i} - \gamma _{j}\right) \right) ^{2}}{\varrho _{ij}^{2}} . \end{aligned}$$

The formula Eq. (4) means that the greater $\gamma _{j}$, the less partons are drawn away from $j$-th country (in our case from Russia); and the greater $\gamma _{i}$, the more $i$-th country attracts partons. Further, the less the Kolmogorov distance between $i$-th and $j$-th countries, the more it facilitates SISM to $i$-th country.

The empirical relationship between quantities $\hat{\mathsf {P}}^{*}$ and ${\mathsf {P}}^{*}$ bear a linear character and can perhaps be expressed by the following regression equation

$$\begin{aligned} \hat{\mathsf {P}}^{*} = 0.004 {\mathsf {P}}^{*} \quad (r^{2}=0{.}744, \quad p=0{.}0000). \end{aligned}$$

Thus, as the statistical analysis has shown, the distribution of partons’ affiliations is significantly connected with ${\mathsf {P}}^{*}$, and hence, with the distribution of citations between countries, and with the Kolmogorov distance between countries. This implies that directions of SISM are determined by differences in scientific impacts between receiver and donor countries.

The advantage of the proposed model is that it accurately describes the relatively complex phenomenon of SISM, and also that Eq. (4) allows for direct interpretation.

## Conclusions

The SISM model does not pretend to represent all the institutional and social circumstances of scientific production. However, it helps to cancel the variety of notions used to describe ISM. Instead of studying separately ‘brain drain’, ‘brain circulation’ or ‘brain gain’, the approach of SISM enables to construct the integral model to describe authors’ localization in the space of affiliation, which is a social space at the same time.

The concept of an affiliation event puts into a precise mathematical form the initially rather vague representation of multiple affiliations. The elements of an exhaustive description of the individual author (and its development in time) are not contained within the conceptual scheme of the SISM model. The idea of the SISM model is that partons are the elementary ingredients of multiple affiliations, and the authors are just bundles of partons. This is not to say that partons are more fundamental than authors. In this paper, we focus on partons, not because they are necessarily more fundamental, but because what we know about partons is more certain. The SISM approach, including the concept of partons, represents an attempt to express the current composition of the scientific labor market using bibliometric methods. In particular, this encompasses the predominance of various forms of part-time, temporary and remote employment of scientists.

Countries with developed science gain the labor of scientists from less advanced countries in the form of temporary, part-time and remote work, which takes the form of SISM. This blurs the national affiliation of successful scientists and generates a virtual international market of scientific labor. Analysis of the Russian case in the space of affiliations clearly outlines the country’s position in the system of global science: between East and West, between developed and newly-developing sciences. The distribution of partons in the space of affiliations is acutely sloped: G7 countries and Switzerland attract a disproportionate number of partons compared to Japan, China, South Korea, Brazil and India. The configuration in the space of affiliations that has formed historically can serve as a predictor of the subsequent development of science in Russia.

Study of SISM enables us to establish the specifics of a country’s involvement in the international division of scientific labor and to determine its position in it. Constructing spaces of affiliations for each country opens up new possibilities for studying the virtual international market of scientific labor. Each country can be represented in the form of a subspace of affiliations, which also represents the country’s position in the global scientific space. Our proposed model of SISM gives a quantitative interpretation of the distribution of partons between countries. It turns out that the number of partons in each country can be explained by the difference between the ‘potential’ of the host country and the donor country (i.e. Russia). If the potential of the ‘attraction’ (the country’s share in total NC) is more than the potential of the ‘push’ (Russia’s share in total NC), then this increases the flow of partons, and vice versa. Moreover, the number of partons associated with a given country strongly depends on the bibliometric distance between Russia and that country. Thus, the strength of the flow of partons is determined not by the absolute values of the host country’s bibliometric indicators, but by its differences from Russia as a donor country.

## References

[CR1] Ackers L, Gill B (2008) Moving people and knowledge scientific mobility in an enlarging European Union. Edward Elgar Publishing Ltd., Cheltenham. http://www.e-elgar.com/shop/moving-people-and-knowledge

[CR2] Andújar I, Cañibano C, Fernández-Zubieta A (2015). International stays abroad, collaborations and the return of Spanish researchers. Sci Technol Soc.

[CR3] Archibugi D, Filippetti A (eds) (2015) The handbook of global science, technology, and innovation. Wiley, Chichester. doi:10.1002/9781118739044

[CR4] Arvizu DE, Bowen RM (eds) (2014) National Science Board. 2014. Science and Engineering Indicators 2014. National Science Foundation, Arlington, VA. http://www.nsf.gov/statistics/seind14/

[CR5] Barré R, Meyer JB, Vink D, Hernandez V (2003) Diasporas scientifiques: comment les pays en développement peuvent-ils tirer parti de leurs chercheurs et de leurs ingénieurs expatriés?. IRD Éditions, Paris

[CR6] Beine M, Docquier F, Rapoport H (2008). Brain drain and human capital formation in developing countries: winners and losers. Econ J.

[CR7] Boring P, Flanagan K, Gagliardi D, Kaloudis A, Karakasidou A (2015). International mobility: findings from a survey of researchers in the EU. Sci Public Policy.

[CR8] Borjas GJ, Doran KB (2012). The collapse of the soviet union and the productivity of american mathematicians*. Q J Econ.

[CR9] Borjas GJ, Doran KB (2012). The collapse of the Soviet Union and the productivity of American mathematicians. Q J Econ.

[CR10] Borovkov AA (2013) Sequences of dependent trials. Markov Chains, Springer, London, pp 386–446. Universitext. doi:10.1007/978-1-4471-5201-9_13

[CR11] Bourdieu P (1985). The social space and the genesis of groups. Theory Soc.

[CR12] Bourdieu P (1989). Social space and symbolic power. Sociol Theory.

[CR13] Boussyguine V (2005) La science en Russie: La nouvelle organisation de la recherche. Éditions L’Harmattan, Paris. http://www.archambault.ca/boussyguine-vladislav-science-en-russie-lala-nouvelle-organisation-de-la-recherche-ACH002855223-fr-pr

[CR14] Cañibano C, Javier Otamendi F, Solis F (2011). International temporary mobility of researchers: a cross-discipline study. Scientometrics.

[CR15] Cañibano C, Bozeman B (2009). Curriculum vitae method in science policy and research evaluation: the state-of-the-art. Res Eval.

[CR16] Charum J, Meyer JB (eds) (1996) International scientific migrations today: new perspectives. Colloques et Séminaires, IRD, Paris. http://www.documentation.ird.fr/hor/fdi:010022327

[CR17] Chikanda A, Crush J, Walton-Roberts M (2016). Diasporas, development and governance, global migration issues.

[CR18] Conchi S, Michels C (2014) Scientific mobility: an analysis of Germany, Austria, France and Great Britain. Fraunhofer ISI discussion papers innovation systems and policy analysis 41, Fraunhofer ISI, Karlsruhe. http://hdl.handle.net/10419/94371

[CR19] De Filippo D, Casado ES, Gómez I (2009). Quantitative and qualitative approaches, to the study of mobility and scientific performance: a case study of a Spanish university. Res Eval.

[CR20] Deville P, Wang D, Sinatra R, Song C, Blondel VD, Barabási AL (2014). Career on the move: geography, stratification, and scientific impact. Sci Rep.

[CR21] Deville P, Wang D, Sinatra R, Song C, Blondel VD, Barabási AL (2014). Career on the move: geography, stratification, and scientific impact. Sci Rep.

[CR22] Ding WW, Levin SG, Stephan PE, Winkler AE (2010). The impact of information technology on academic scientists’ productivity and collaboration patterns. Manag Sci.

[CR23] Docquier F, Rapoport H (2012). Globalization, brain drain, and development. J Econ Lit.

[CR24] Dubois P, Rochet JC, Schlenker JM (2014). Productivity and mobility in academic research: evidence from mathematicians. Scientometrics.

[CR25] Edler J, Fier H, Grimpe C (2011). International scientist mobility and the locus of knowledge and technology transfer. Res Policy.

[CR26] Fernández-Zubieta A, Geuna A, Lawson C (2015) What do we know of the mobility of research scientists and impact on scientific production. In: Global mobility of research scientists. Elsevier BV, Amsterdam, pp 1–33. doi:10.1016/B978-0-12-801396-0.00001-6

[CR27] Flanagan K (2015) International mobility of scientists. In: Archibugi D, Filippetti A (eds) The handbook of global science, technology, and innovation. Wiley, Chichester, pp 364–381. doi:10.1002/9781118739044.ch17

[CR28] Furukawa T, Shirakawa N, Okuwada K (2011). Quantitative analysis of collaborative and mobility networks. Scientometrics.

[CR29] Furukawa T, Shirakawa N, Okuwada K, Sasaki K (2012). International mobility of researchers in robotics, computer vision and electron devices: a quantitative and comparative analysis. Scientometrics.

[CR30] Gaillard J, Gaillard AM (1997). Introduction: the international mobility of brains: exodus or circulation?. Sci Technol Soc.

[CR31] Ganguli I (2015). Immigration and ideas: what did russian scientists “bring” to the United States?. J Labor Econ.

[CR32] Gargiulo F, Carletti T (2014). Driving forces of researchers mobility. Sci Rep.

[CR33] Gerber TP, Yarsike Ball D (2009). Scientists in a changed institutional environment: subjective adaptation and social responsibility norms in Russia. Soc Stud Sci.

[CR34] Geuna A (2015) Global mobility of research scientists: the economics of who goes where and why. Elsevier, Boston. doi:10.1016/B978-0-12-801396-0.01001-2

[CR35] Graham LR, Dezhina I (2008) Science in the new Russia: crisis, aid, reform. Indiana University Press, Bloomington. https://lccn.loc.gov/2007047371

[CR36] Harvey WS (2011). British and Indian scientists moving to the United States. Work Occup.

[CR37] Helbing D (2010) Quantitative sociodynamics. Stochastic methods and models of social interaction processes, 2nd edn. Springer, Berlin. doi:10.1007/978-3-642-11546-2

[CR38] Indukaev A, Mogoutov A, Lepinay V (2014) Computer scientists from the former USSR: international mobility patterns and scientific success. In: Proceedings of the 10th Central and Eastern European software engineering conference in Russia, ACM, New York, NY, CEE – SECR’14, pp 7:1–7:9. doi:10.1145/2687233.2687257

[CR39] Ivakhnyuk I, Gmaj K, Iglicka K (2006). Brain drain from Russia: in search for a solution. Brain drain or brain gain—a global dilemma.

[CR40] Jacod J, Shiryaev AN (2003) Skorokhod topology and convergence of processes. In: Limit theorems for stochastic processes. Springer, Berlin, pp 324–388. doi:10.1007/978-3-662-05265-5_6

[CR41] Jonkers K (2010) Mobility, migration, and the Chinese scientific research system. No. 50 in Routledge contemporary China series, Routledge, London, 00016

[CR42] Jonkers K, Cruz-Castro L (2013). Research upon return: the effect of international mobility on scientific ties, production and impact. Res Policy.

[CR43] Jöns H (2009). “Brain circulation” and transnational knowledge networks: studying long-term effects of academic mobility to Germany, 1954–2000. Glob Netw.

[CR44] Korobkov AV, Zaionchkovskaia ZA (2012). Russian brain drain: myths v. reality.. Communist Post Communist Stud.

[CR45] Krause J, Lindqvist CM, Mele S (2007) Quantitative study of the geographical distribution of the authorship of high-energy physics journals. Tech. Rep. CERN-OPEN-2007-014, CERN, Geneva. http://cds.cern.ch/record/1033099

[CR46] Latova NV, Savinkov VI (2012). The influence of academic migration on the intellectual potential of Russia. Eur J Educ.

[CR47] Laudel G (2005). Migration currents among the scientific elite. Minerva.

[CR48] Lawson C, Shibayama S (2013) Temporary mobility—a policy for academic career development. SSRN Scholarly Paper ID 2257889, Social Science Research Network, Rochester, NY. http://papers.ssrn.com/abstract=2257889

[CR49] Li W, Yu W, Sadowski-Smith C, Wang H (2015). Intellectual migration and brain circulation: conceptual framework and empirical evidence. J Chin Overseas.

[CR50] Lu X, Zhang W (2015). The reversed brain drain: a mixed-method study of the reversed migration of Chinese overseas scientists. Sci Technol Soc.

[CR51] Marmolejo-Leyva R, Perez-Angon MA, Russell JM (2015). Mobility and international collaboration: case of the Mexican scientific diaspora. PLoS One.

[CR52] Meyer JB, Miao FW, Zhao Y (2016) Visualizing the diaspora: new options. In: Chikanda A, Crush J, Walton-Roberts M (eds) Diasporas, development and governance. Springer, Cham, pp 205–220. doi:10.1007/978-3-319-22165-6_13

[CR53] Mingers J, Leydesdorff L (2015). A review of theory and practice in scientometrics. Eur J Oper Res.

[CR54] Moed HF, Halevi G (2014). A bibliometric approach to tracking international scientific migration. Scientometrics.

[CR55] Moed HF, Aisati M, Plume A (2013). Studying scientific migration in Scopus. Scientometrics.

[CR56] OECD (2013) OECD science, technology and industry scoreboard 2013: innovation for growth. OECD Publishing, Paris. doi:10.1787/sti_scoreboard-2013-en

[CR57] Pan RK, Kaski K, Fortunato S (2012). World citation and collaboration networks: uncovering the role of geography in science. Sci Rep.

[CR58] Pierson AS, Cotgreave P (2000). Citation figures suggest that the UK brain drain is a genuine problem. Nature.

[CR59] Quayle M, Greer M (2014). Mapping the state of the field of social psychology in Africa and patterns of collaboration between African and international social psychologists. Int J Psychol.

[CR60] Rachev ST, Klebanov LB, Stoyanov SV, Fabozzi FJ (2013) Probability distances and probability metrics: definitions. In: The methods of distances in the theory of probability and statistics. Springer, New York, pp 11–31. doi:10.1007/978-1-4614-4869-3_2

[CR61] Report of a committee appointed by the Royal Society (1963). The emigration of scientists from the United Kingdom: report of a committee appointed by the Royal Society. Minerva.

[CR62] Roberge G, Campbell D (2012) Canadian researchers migration analysis based on Scopus Author IDs. In: 17th international conference on science and technology indicators (STI 2012), Montreal, vol 2, pp 884–885. http://sticonference.org/Proceedings/vol2/Roberge_Canadian_884

[CR63] Sandström U (2009). Combining curriculum vitae and bibliometric analysis: mobility, gender and research performance. Res Eval.

[CR64] Saxenian A (2005). From brain drain to brain circulation: transnational communities and regional upgrading in India and China. Stud Comp Int Dev.

[CR65] Seguin B (2006). Scientific diasporas. Science.

[CR66] Shkolnikov VD (1994). Scientific bodies in motion: the domestic and international consequences of the current and emergent brain drain from the former USSR.

[CR67] Shmatko NA, Katchanov YL (2016) Professional careers and mobility of Russian doctorate holders. In: Auriol L, Gokhberg LM, Shmatko NA (eds) The science and technology labor force. The Value of Doctorate Holders and Development of Professional Careers, Science, Technology and Innovation Studies. Springer, Berlin, pp 179–204. doi:10.1007/978-3-319-27210-8

[CR68] Solimano A (ed) (2008) The international mobility of talent: types, causes, and development impact. Oxford University Press, Oxford. doi:10.1093/acprof:oso/9780199532605.001.0001

[CR69] State B, Rodriguez M, Helbing D, Zagheni E (2014) Migration of professionals to the U.S. In: Aiello LM, McFarland D (eds) Social informatics, lecture notes in computer science, vol 8851. Springer, Cham, pp 531–543. doi:10.1007/978-3-319-13734-6_37

[CR70] Stephan PE (2012) How economics shapes science. Harvard University Press, Cambridge. https://lccn.loc.gov/2011013433

[CR71] Takane Y, Young FW, de Leeuw J (1977). Nonmetric individual differences multidimensional scaling: an alternating least squares method with optimal scaling features. Psychometrika.

[CR72] Van Noorden R (2012). Global mobility: science on the move. Nature.

[CR73] Veugelers R, Van Bouwel L (2015) Destinations of mobile european researchers: Europe versus the United States, Chapter 8. Academic Press, San Diego, pp 215–237. doi:10.1016/B978-0-12-801396-0.00008-9

[CR74] Wang X, Mao W, Wang C, Peng L, Hou H (2013). Chinese elite brain drain to USA: an investigation of 100 United States national universities. Scientometrics.

[CR75] Weidlich W (2006) Group Dynamics: the rise and fall of interacting social groups. In: Sociodynamics: a systemic approach to mathematical modelling in the social sciences. Dover Publications, Mineola, pp 115–147. http://store.doverpublications.com/0486450279.html

[CR76] Weidlich W, Haag G (1988) Concepts of the dynamic migration model. In: Weidlich W, Haag G (eds) Interregional migration: dynamic theory and comparative analysis. Springer, Berlin, pp 9–20. doi:10.1007/978-3-642-73049-8

[CR77] Woolley R, Turpin T (2009). CV analysis as a complementary methodological approach: investigating the mobility of Australian scientists. Res Eval.

